# Effectiveness of human-origin *Lactobacillus plantarum* PL-02 in improving muscle mass, exercise performance and anti-fatigue

**DOI:** 10.1038/s41598-021-98958-x

**Published:** 2021-09-30

**Authors:** Mon-Chien Lee, Yi-Ju Hsu, Hsieh‐Hsun Ho, Yi‐Wei Kuo, Wen-Yang Lin, Shin-Yu Tsai, Wei-Ling Chen, Che-Li Lin, Chi-Chang Huang

**Affiliations:** 1 Graduate Institute of Sports Science, National Taiwan Sport University, Taoyuan City, 333325 Taiwan; 2 Research and Development Department, Bioflag Biotech Co, Ltd, Tainan, Taiwan; 3 Department of Sports Training Science-Athletics, National Taiwan Sport University, Taoyuan City, 333325 Taiwan; 4 Department of Orthopedics, School of Medicine, College of Medicine, Taipei Medical University, Taipei City, 11031 Taiwan

**Keywords:** Nutrition, Applied microbiology

## Abstract

Gut microbiota is very important for energy metabolism and regulation, which in turn affect the health and physiological functions of the host, and provide energy required for exercise. Supplementation with probiotics may be one of the ways to change the gut microbiota. In recent years, many studies have shown that probiotic supplementation can effectively improve sports performance. In this study, we screened *Lactobacillus plantarum* (PL-02), a probiotic of human-origin, from the intestines of 2008 Olympic women's 48 kg weightlifting gold medalist and explored the role of PL-02 in improved exercise endurance performance, reduced fatigue biochemical parameters, and changes in body composition. Male Institute of Cancer Research (ICR) mice were assigned to 0, 2.05 × 10^9^, 4.10 × 10^9^ and 1.03 × 10^10^ CFU/kg/day groups and were fed by oral gavage once daily for 4 weeks. The results showed that 4 weeks of PL-02 supplementation could significantly increase muscle mass, muscle strength and endurance performance, and hepatic and muscular glycogen storage. Furthermore, PL-02 could significantly decrease lactate, blood urea nitrogen (BUN), ammonia, and creatine kinase (CK) levels after exercise (*p* < 0.05). We believe that PL-02 can be used as a supplement to improve exercise performance and for its anti-fatigue effect.

## Introduction

Exercise-induced fatigue can be roughly divided into central fatigue and peripheral fatigue, which is a common and complex multi-dimensional symptom^1^. Among them, peripheral fatigue generally refers to long-term or high-intensity exercise; the body is unable to provide or maintain the required energy load, resulting in decreased performance and producing fatigue^2^. During exercise, glycogen from the liver and muscles is metabolized into glucose by the phosphocreatine system, and glucose is further metabolized to meet higher energy requirements^3^. When the energy consumption exceeds the supply and demand, the by-products that cause fatigue will increase, especially in the muscles^4^. Due to the consumption of creatine phosphate, the accumulation of neuromuscular signaling implants, glycogen, and internal metabolites (including lactic acid, inorganic phosphorus, and ammonia), it is difficult for the muscles to maintain continuous contraction^4,5^. These metabolites can cause muscle fatigue through intracellular acidosis in the body^6^. Therefore, to recover from fatigue caused by exercise, it is necessary to repair damage to the body and eliminate the metabolites accumulated from exercise. In addition to scientific training methods and nutritional supplementation for those who engage in sports, in recent years, views on gut microbiota relative to energy regulation have gradually attracted attention^7^.

The gut microbiota contains trillions of different microbial communities. Its rich and diverse ecosystem interacts with the host, which is the key to metabolism and is believed to play a key role in the overall health and disease of the host^8^. By promoting the development and maturation of intestinal epithelial cells, it activates the intestinal barrier function and prevents the colonization of pathogenic bacteria to maintain the health of the host^9^. The most important source of nutrition for the gut microbiota is indigestible carbohydrates in the diet^10^. Promoting the digestion, absorption, and fermentation of them to produce short-chain fatty acids (SCFA), such as acetate, propionate, and butyrate, as well as H2 and CO2 gases, ammonia, amines, phenols, and energy, helps to maintain cell function^11^. Among them, propionic acid is used in gluconeogenic liver cells, while butyrate is affected by the acetoacetyl-CoA produced by fatty acid β-oxidation (FAO), which enters the trichloroacetic acid cycle to produce adenosine triphosphate (ATP) and CO_2_^12^. In addition to the induction of PGC-1α gene expression in skeletal muscle and brown adipose tissue (BAT)^13^ and the activation of the activated protein kinase-acetyl-CoA carboxylase (AMPK-ACC) pathway to improve respiratory capacity, FAO is a key regulator of energy production and mitochondrial function. It is regarded as one of the important factors for improving exercise performance^14^. In recent years, a study has shown that through the action of the intestinal axis, gut microbiota seems to have an impact on muscle mass^15^.

There are many factors that affect changes in the gut microbiota, including age, gender, antibiotics, diet, and exercise^16^. However, supplementation with probiotics is also one of the feasible methods. The modern definition of probiotic was put forward as an individual or mixed culture of bacteria, through ingestion, it can be colonized in the intestinal tract to increase the diversity and richness ratio of good bacteria in the intestine, thereby increasing the host's energy metabolism and promoting health^17^. Different strains have different effects, and the most of the common probiotics are *Bifidobacterium* or *Lactobacillus*^18^. Among them, *Lactobacillus plantarum* (*L. plantarum*) is a Gram-positive species and belongs to the *Lactobacillus* spp. Many studies in the past have shown that it has anti-inflammatory^19^, antioxidant^20^, and other effects. In addition, *L. plantarum* can help bacteria use lactic acid, firstly producing acetyl-CoA from lactic acid. Two molecules of acetyl-CoA are combined and reduced to produce butyryl-CoA, then converts butyryl-CoA to butyryl-phosphate by using the phosphotransbutyrylase enzyme. Butyryl-phosphate is converted into butyrate by using butyrate kinase and releases ATP, is regarded as an important factor in reducing exercise fatigue^21^. Although it has been confirmed in animal^22^ and human^23^ experiments that *L. plantarum* could improve exercise performance and reduce fatigue biochemical indicators, the strain was selected from kimchi.

Past study has found that athletes have a higher gut microbial diversity^24^. Exercise would cause changes in the gut microbiota to increase diversity and abundance, improve amino acid utilization, carbohydrate metabolism and fecal metabolite production to promote energy collection and utilization^25^. In recent years, our country’s weightlifting program has continuously achieved good results in various international competitions. In addition to professional skills and explosive power, the training of weightlifters also has many training subjects about physical fitness and endurance. Therefore, in the current study, we screened edible strains of *L. plantarum* (PL-02), which is a human-origin probiotic, from the intestines of Olympic gold medalists and explored the effect of PL-02 in terms of improving exercise endurance, reducing fatigue biochemical parameters, and changing body composition. In addition, we further analyzed the gut microbiota, hoping to explore the possible mechanism of probiotics against fatigue and to confirm its safety.

## Materials and methods

### Sample preparation

We screened *L. plantarum* (PL-02) from the fecal of Wei-Ling Chen, who was famous for the winner of 2008 summer Olympics weightlifting women's 48 kg gold medal winner, and the stool preservation kit was used to collect the fecal specimen. We used the Glucose-molasses medium (GMM) to culture the specimen for 1–4 days at 37 degrees under three different oxygen conditions (Aerobic, facultative anaerobic and obligate anaerobic). The cultured intestinal microflora extracts DNA by 16S rDNA sequencing, and sequence the microbiota of the fecal specimen, and selected the *L. plantarum* (PL-02). The Food Industry Research and Development Institute (Hsinchu, Taiwan) has confirmed that the isolate is *L. plantarum*. The dry product of PL-02 was prepared and provided by Bioflag Biotech Co, Ltd. (Tainan, Taiwan). The viable number of PL-02 cells was 1.07 × 10^11^ CFU/g. Before consumption, the powder was suspended in phosphate buffered saline (PBS, pH 7.2).

### Animals and experimental design

In the current study, the microbial strain was isolated from one of the Co-authors (Wei-Ling Chen) of the Manuscript, all experimental used the animal model for research, and the methods followed the Guideline for the Care and Use of Laboratory Animals of Council of Agriculture, Executive Yuan, Taiwan and ARRIVE guidelines. The experiments were approved by the Institutional Animal Care and Use Committee (IACUC) of National Taiwan Sport University (approval number: IACUC-10904). All the mice were kept in a light–dark cycle for 12 h at room temperature (23 ± 2 °C) and 50–60% humidity and were provided with reverse osmosis (RO) water and a standard daily diet (No. 5001; PMI Nutrition International, Brentwood, MO, USA) ad libitum. Since probiotics are used in humans at a dose of 1 × 10^10^ live bacteria per day^26^, the human body surface area is converted from mouse equivalent dose (HED) to a mouse dose. Forty 6-week-old male ICR mice were purchased from BioLASCO (Yi-Lan, Taiwan). After a 2-week acclimation period, all the mice were divided into four groups (n = 10 in each group), and they were administered by gavage once a day for 4 weeks: (1): vehicle group (0 CFU/kg); (2): PL-02-1X group (2.05 × 10^9^ CFU/kg); (3): PL-02-2X group (4.10 × 10^9^ CFU/kg), and (4): PL-02-5X group (1.03 × 10^10^ CFU/kg). All groups were given the same volume of PBS or supplements, and the dose of PL-02 was determined according to the body weight of each mouse.

### Forelimb grip strength

As previously described, a low-force test system (Model-RX-5, Aikoh Engineering, Nagoya, Japan) with a tension rod (diameter 2 mm, length 7.5 cm) and a force sensor was used to measure the grip of mice^27^.

### Exercise performance test

In order to understand the effect of PL-02 on improving exercise endurance performance, we conducted a swimming exhaustion test. The tails of all mice were attached with 5% body weight, and the duration of such swimming was recorded. The test mice were then forced to swim until they lost coordinated movement or could not return to the surface within 7 s, as described previously^28^.

### Determination of fatigue-associated biochemical variables

In order to understand the effect of PL-02 supplementation on fatigue-related indicators and physiological adaptation after exercise, we collected blood samples after swimming for 10 min and resting for 20 min to analyze blood lactic acid, blood ammonia, and glucose. After 90 min of prolonged exercise and 60 min of rest, we immediately evaluated other variables such as blood urine nitrogen (BUN) and creatine kinase (CK). All the mice were ensured for fasting before all exercise tests to ensure conditional control, as previously described^28^. The sample was centrifuged at 1500×*g* at 4 °C for 10 min, and the serum was collected and measured by an automatic analyzer (Hitachi, Tokyo, Japan, Hitachi 7060).

### Clinical biochemical profiles

At the end of the study, all the mice were euthanized by 95% CO2 asphyxiation one hour after the last treatment and blood was obtained by cardiac puncture. Serum was collected after centrifugation, and biochemical indexes were assessed by Hitachi 7060 autoanalyzer. Levels of glucose (GLU), albumin (ALB), total protein (TP), creatine kinase (CK), aspartate aminotransferase (AST), alanine transaminase (ALT), total cholesterol (TC), triglycerides (TG), blood urea nitrogen (BUN), creatinine (CREA), and uric acid (UA) were measured.

### Body composition, glycogen content, and histopathology

After the mice were euthanized, the liver, muscle, kidney, heart, lung, epididymal fat pad (EFP), and brown adipose tissue (BAT) were accurately excised and weighed. The organs were carefully removed, chopped, and fixed in 10% formalin. The tissue was embedded in paraffin and cut into 4 μm-thick sections for morphological and pathological evaluation. As mentioned earlier, the sections were stained with hematoxylin and eosin (H&E) and inspected under an optical microscope equipped with a charge-coupled device (CCD) camera (BX-51, Olympus, Tokyo, Japan)^27^. Parts of the muscle and liver tissues were stored in liquid nitrogen for glycogen content analysis, as previously described^29^.

### Bacterial DNA extraction and 16S rRNA sequencing

According to the method previously used in our laboratory, immediately after the euthanasia of the mice, the collected samples were stored at -80 °C for DNA extraction. The detailed procedures for sample extraction, preparation, and analysis have been described previously^30^.

### Statistical analysis

All data are expressed as mean ± SD. The statistical analysis was performed in SAS 9.0 (SAS Inst., Cary, NC, USA). Using Duncan's post hoc test, multiple group comparisons were analyzed by one-way analysis of variance (ANOVA). Cochran-Armitage test is used for dose–effect trend analysis. Statistical significance was set at *p* < 0.05.

Statistical analysis was performed using the CLC Microbial Genomics Module (v10.1.1). Alpha diversity was measured using Shannon index, which calculates the overall diversity of each group including the number of observed species (richness) and how evenly of observed taxonomic (evenness). Beta diversity was measured using PCoA-Unweighted UniFrac, which determines the difference of microbial composition between groups. Alpha and beta diversity plots were utilized by the R language (v4.0.2) with ggplot2 package. Comparison of different groups was performed by t test with two-tailed and PERMANOVA analysis. A *p* value less than 0.05 were considered statistically significant.

## Result

### General characteristics of mice with PL-02 supplementation for four weeks

As shown on Table 1, after four consecutive weeks of supplementation with PL-02, the weight of each group of mice showed stable growth; there was no significant difference between the groups, and there were no significant differences in the food intake and water intake of each group of mice. However, supplementation with PL-02 could effectively only improve skeletal muscle mass, which in the vehicle, PL-02-1X, PL-02-2X, and PL-02-5X groups was 0.37 ± 0.04, 0.39 ± 0.02, 0.40 ± 0.03, and 0.41 ± 0.02 (g), respectively. Compared with the placebo group, only the PL-02-5X group significantly increased by 1.10 -fold (*p* = 0.0028). But there is still a significant dose dependence, *p* = 0.0015. As the tissue weight is affected by the weight difference, we divided the tissue weight by the relative percentage of body weight and found that the relative skeletal muscles weight in the vehicle, PL-02-1X, PL-02-2X, and PL-02-5X groups was 1.00 ± 0.04, 1.05 ± 0.02, 1.06 ± 0.03, and 1.10 ± 0.02 (g), respectively. Compared with the placebo group, the PL-02-1X, PL-02-2X, and PL-02-5X groups were significantly increased relative skeletal muscles weight by 1.05 -fold (*p* = 0.0014), 1.06-fold (*p* = 0.0001), 1.11-fold (*p* < 0.0001), respectively. The effect of PL-02 supplementation on relative muscle weight was dose dependent (*p* < 0.0001).

**Table 1:** Effect of PL-02 supplementation on various parameters.

Characteristic	Vehicle (PBS)	PL-02-1X	PL-02-2X	PL-02-5X	Trend analysis
Initial BW (g)	34.8 ± 1.1	34.6 ± 1.3	34.5 ± 1.4	34.6 ± 1.0	0.4507
Final BW (g)	37.3 ± 2.8	37.5 ± 1.4	37.4 ± 1.4	37.2 ± 1.6	0.8624
Water intake (mL/mouse/day)	8.1 ± 2.3	8.1 ± 2.2	8.2 ± 2.3	8.1 ± 1.4	0.2061
Food intake (g/mouse/day)	6.8 ± 1.3	6.7 ± 1.3	6.8 ± 1.3	6.4 ± 1.2	0.1453
Liver (g)	2.01 ± 0.12	1.99 ± 0.16	2.00 ± 0.18	2.01 ± 0.12	0.8113
Muscle (g)	0.37 ± 0.04^a^	0.39 ± 0.02^ab^	0.40 ± 0.03^ab^	0.41 ± 0.02^b^	0.0015*
Quadriceps (g)	0.51 ± 0.06	0.53 ± 0.06	0.53 ± 0.04	0.53 ± 0.05	0.5256
Kidney (g)	0.59 ± 0.07	0.60 ± 0.06	0.59 ± 0.05	0.59 ± 0.03	0.9523
Heart (g)	0.19 ± 0.03	0.20 ± 0.02	0.20 ± 0.03	0.19 ± 0.02	0.2786
Lung (g)	0.25 ± 0.03	0.24 ± 0.05	0.25 ± 0.01	0.24 ± 0.03	0.6396
EFP (g)	0.36 ± 0.04	0.35 ± 0.10	0.36 ± 0.15	0.34 ± 0.12	0.3592
BAT (g)	0.11 ± 0.02	0.11 ± 0.02	0.11 ± 0.03	0.10 ± 0.01	0.5523
Cecum (g)	0.83 ± 0.08	0.83 ± 0.16	0.84 ± 0.08	0.84 ± 0.09	0.6607
*Relative liver weight (%)	5.39 ± 0.14	5.31 ± 0.25	5.35 ± 0.31	5.40 ± 0.13	0.9568
Relative muscle weight (%)	1.00 ± 0.04^a^	1.05 ± 0.02^b^	1.06 ± 0.03^b^	1.10 ± 0.02^c^	< 0.0001*
Relative quadriceps weight (%)	1.37 ± 0.06	1.40 ± 0.11	1.41 ± 0.05	1.43 ± 0.06	0.0785
Relative kidney weight (%)	1.58 ± 0.07	1.60 ± 0.11	1.58 ± 0.08	1.58 ± 0.02	0.8170
Relative heart weight (%)	0.51 ± 0.04	0.52 ± 0.03	0.53 ± 0.05	0.52 ± 0.02	0.1083
Relative lung weight (%)	0.66 ± 0.04	0.63 ± 0.12	0.67 ± 0.02	0.64 ± 0.06	0.5418
Relative EFP weight (%)	0.96 ± 0.05	0.94 ± 0.23	0.96 ± 0.37	0.91 ± 0.29	0.2944
Relative BAT weight (%)	0.28 ± 0.05	0.30 ± 0.04	0.29 ± 0.02	0.27 ± 0.03	0.4544
Relative cecum weight (%)	2.21 ± 0.07	2.21 ± 0.35	2.25 ± 0.15	2.25 ± 0.14	0.6468

Data are expressed as mean ± SD (*n* = 10 mice per group).

*EFP* epididymal fat pad, *BAT* brown adipose tissue.

*Relative to body weight.

### Effect of PL-02 supplementation on exercise performance

**Figure 1:**
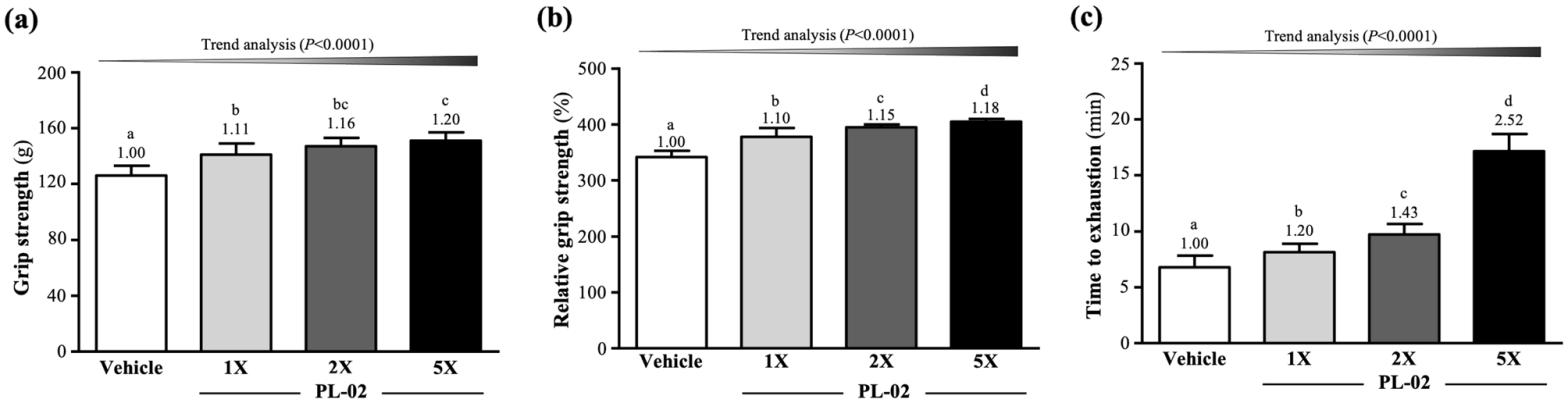
Effect of 4 weeks of PL-02 supplementation on (**a**) absolute forelimb grip strength, (**b**) forelimb grip strength (%) relative to body weight and (**c**) exhaustive swim time. Data are expressed as mean ± SD for n = 10 mice per group. Different superscript letters (a, b, c, d) indicate significant difference at *p* < 0.05.

On grip strength, after 4 weeks of PL-02 supplementation, the mean forelimb grip strengths of mice in the vehicle, PL-02-1X, PL-02-2X, and PL-02-5X groups were 126 ± 7, 141 ± 8, 147 ± 6, and 151 ± 6 g, respectively (Fig. 1a) The PL-02-1X, PL-02-2X, and PL-02-5X groups were significantly higher than the vehicle group by 1.11-fold (*p* < 0.0001), 1.16-fold (*p* < 0.0001), and 1.20-fold (*p* < 0.0001), respectively. Relative grip strength (%), normalized to body weight, was also significantly higher in groups with PL-02 supplementation (Fig. 1b). The effects of PL-02 supplementation on absolute and relative grip strength were dose dependent (*p* < 0.0001).

After four weeks of PL-02 supplementation, the exhaustive swim times in the vehicle, PL-02-1X, PL-02-2X, and PL-02-5X groups were 6.81 ± 1.04, 8.16 ± 0.75, 9.74 ± 0.93, and 17.16 ± 1.53 min, respectively. The average exhaustive swim time of the PL-02-1X, PL-02-2X, and PL-02-5X groups was significantly increased by 1.20-fold (*p* = 0.0092), 1.43-fold (*p* < 0.0001), and 2.52-fold (*p* < 0.0001), respectively, as compared to the vehicle group. The trend analyses showed that the effect of PL-02 supplementation on maximum swim time was dose dependent (*p* < 0.0001) (Fig. 1c).

### Effect of PL-02 supplementation on serum lactate levels after the 10-min swim test

After 4 weeks of supplementation with PL-02, all the mice were subjected to the 10-min swimming test to evaluate the levels of lactate (Table 2). Before swimming, there was no significant difference in blood lactate levels between groups. The serum lactate levels of mice in the vehicle, PL-02-1X, PL-02-2X, and PL-02-5X groups were 7.78 ± 0.70, 5.67 ± 0.78, 4.90 ± 0.55 and 4.65 ± 0.52 mmol/L after 10 min of swimming, respectively. Compared with the placebo group, the PL-02-1X, PL-02-2X, and PL-02-5X groups were significantly decreased by 27.16% (*p* < 0.0001), 37.05% (*p* < 0.0001), and 40.26% (*p* < 0.0001), respectively. Based on the serum lactate concentration before and after 10 min of swimming, the lactate production rates were determined to be 2.51 ± 0.22, 1.80 ± 0.08, 1.55 ± 0.09, and 1.46 ± 0.09, respectively, in the vehicle, PL-02-1X, PL-02-2X, and PL-02-5X groups. The PL-02-1X, PL-02-2X, and PL-02-5X groups were significantly lower than vehicle group by 27.99% % (*p* < 0.0001), 38.26% (*p* < 0.0001), and 41.74% (*p* < 0.0001), respectively.

**Table 2:** Effect of PL-02 on lactate levels.

Time point	Vehicle	PL-02-1X	PL-02-2X	PL-02-5X	Trend Analysis
**Lactate (mmol/L)**					
Before swimming (A)	3.15 ± 0.54	3.13 ± 0.36	3.17 ± 0.33	3.19 ± 0.32	0.6067
After swimming (B)	7.78 ± 0.70^c^	5.67 ± 0.78^b^	4.90 ± 0.55^a^	4.65 ± 0.52^a^	< 0.0001*
After a 20 min rest (C)	6.30 ± 0.56^c^	4.58 ± 0.54^b^	3.87 ± 0.42^a^	3.46 ± 0.34^a^	< 0.0001*
**Rate of lactate production and clearance**					
Production rate = B/A	2.51 ± 0.22^c^	1.80 ± 0.08^b^	1.55 ± 0.09^a^	1.46 ± 0.09^a^	< 0.0001*
Clearance rate = (B-C)/B	0.19 ± 0.04^a^	0.19 ± 0.04^a^	0.21 ± 0.04^a^	0.25 ± 0.03^b^	< 0.0001*

Lactate production rate (B/A) was the value of the lactate level after exercise (B) divided by that before exercise (A). Clearance rate (B − C)/B was defined as lactate level after swimming (B) minus that after 20 min rest (C) divided by that after swimming (B). Data are expressed as mean ± SD (*n* = 10 mice per group). Values in the same row with different superscript letters (a, b, c, d) differ significantly, *p* < 0.05.

After 20 min rest following the swimming test, the blood lactate levels in the vehicle, PL-02-1X, PL-02-2X, and PL-02-5X groups were 6.30 ± 0.56, 4.58 ± 0.54, 3.87 ± 0.42, and 3.46 ± 0.34 mmol/L, respectively. This represented a decrease of 27.35% (*p* = 0.0002), 38.61% (*p* < 0.0001), and 45.12% (*p* < 0.0001) in the PL-02-1X, PL-02-2X, and PL-02-5X groups, respectively, compared to the vehicle group. The clearance rate is used to understand the recovery effect of lactate after 10 min of exercise then 20 min of rest, which was determined to be 0.19 ± 0.04, 0.19 ± 0.04, 0.21 ± 0.04, and 0.25 ± 0.03, respectively, in the vehicle, PL-02-1X, PL-02-2X, and PL-02-5X groups. Compared with the placebo group, only the PL-02-5X group significantly increased by 1.34 -fold (*p* = 0.0003). The effect of PL-02 supplementation on serum lactate levels was also dose dependent.

### Effect of PL-02 supplementation on fatigue-related biochemical parameters after the 10-min swim test or a 90 min swim test and 60 min rest

**Figure 2:**
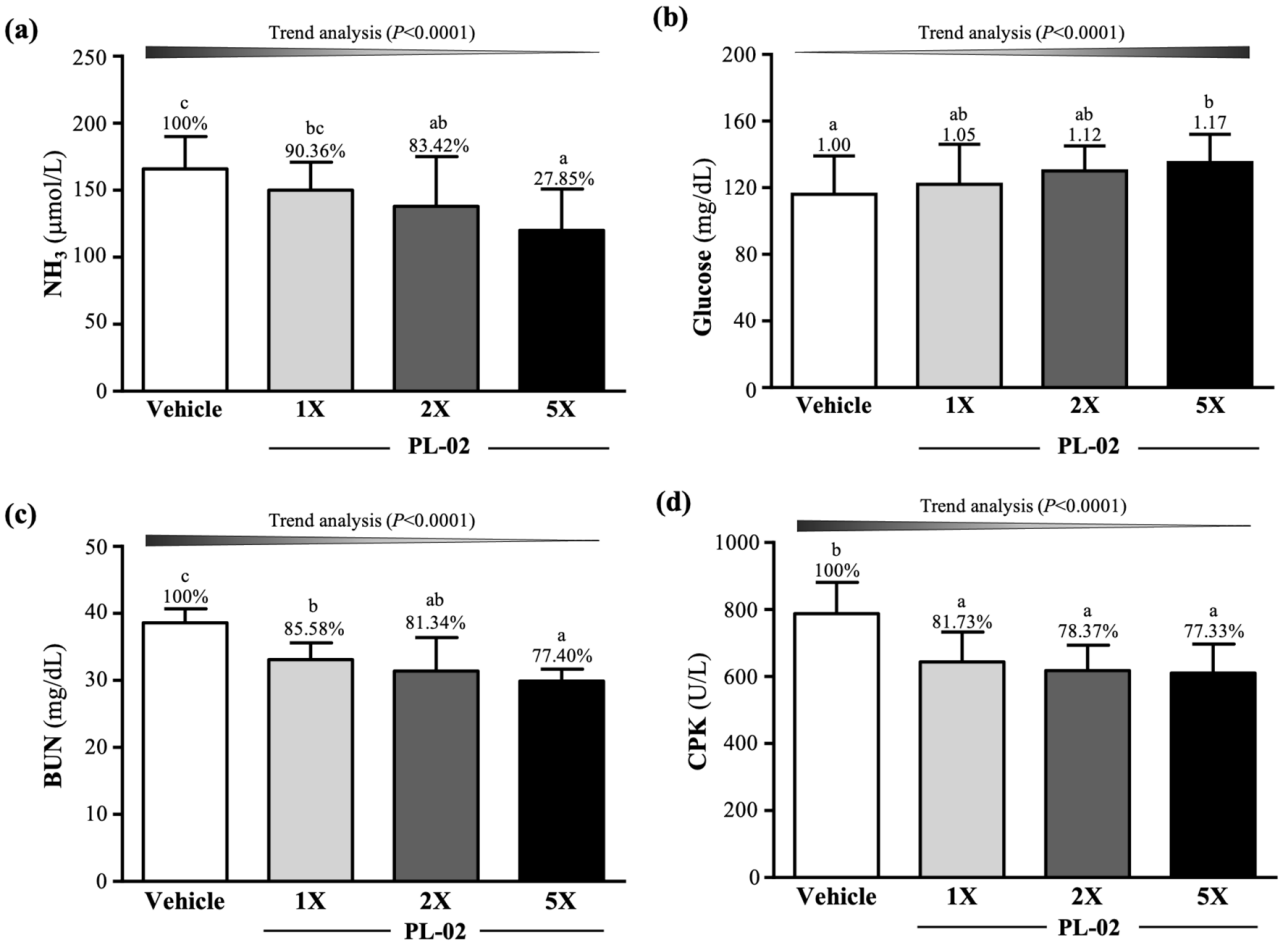
Effect of 4 weeks PL-02 supplementation on (**a**) NH_3_ and (**b**) glucose, (**c**) BUN and (**d**) CK. Data are expressed as mean ± SD for n = 10 mice per group. Different superscript letters (a, b, c) indicate significant difference at *p* < 0.05.

As shown in Fig. 2a, after the 10 min swim test, the serum ammonia levels in the vehicle, PL-02-1X, PL-02-2X, and PL-02-5X groups were 166 ± 24, 150 ± 21, 138 ± 37, and 120 ± 31 µmol/L, respectively. Compared with vehicle group, PL-02-2X and PL-02-5X groups were significantly decreased by 16.58% (*p* = 0.0423) and 27.85% (*p* = 0.0011). The glucose levels in the vehicle, PL-02-1X, PL-02-2X, and PL-02-5X groups were 116 ± 23, 122 ± 23, 130 ± 15, and 135 ± 17 mg/dL. Only PL-02-5X group was significantly greater than vehicle group by 1.17 -fold (*p* = 0.0368) (Fig. 2b). Supplementation with PL-02 could decrease ammonia and improve glucose levels after exercise, both of which have a dose-dependent effect (*p* < 0.0001).

The serum BUN levels were measured 60 min after the 90-min swimming test (Fig. 2c), which were found to be 38.6 ± 2.1, 33.1 ± 2.5, 31.4 ± 5.0, and 29.9 ± 1.8 mg/dL in the mice in the vehicle, PL-02-1X, PL-02-2X, and PL-02-5X groups, respectively. Compared with the vehicle group, the PL-02-1X, PL-02-2X, and PL-02-5X groups were significantly decreased by 14.42% (*p* = 0.0003), 18.66% (*p* < 0.0001), and 22.60% (*p* < 0.0001). The effect of PL-02 supplementation on serum BUN levels was also dose dependent (*p* < 0.0001). An exercise injury index, CK, had a significant difference among the groups after the 90 min swim test and 60 min rest (Fig. 2d). Compared with the vehicle group, the PL-02-1X, PL-02-2X, and PL-02-5X groups were significantly decreased by 18.27% (*p* = 0.0007), 21.63% (*p* < 0.0001), and 22.67% (*p* < 0.0001). The effect of PL-02 supplementation on serum CK levels was also dose dependent (*p* < 0.0001).

### Effect of PL-02 Supplementation on liver and muscle glycogen

**Figure 3:**
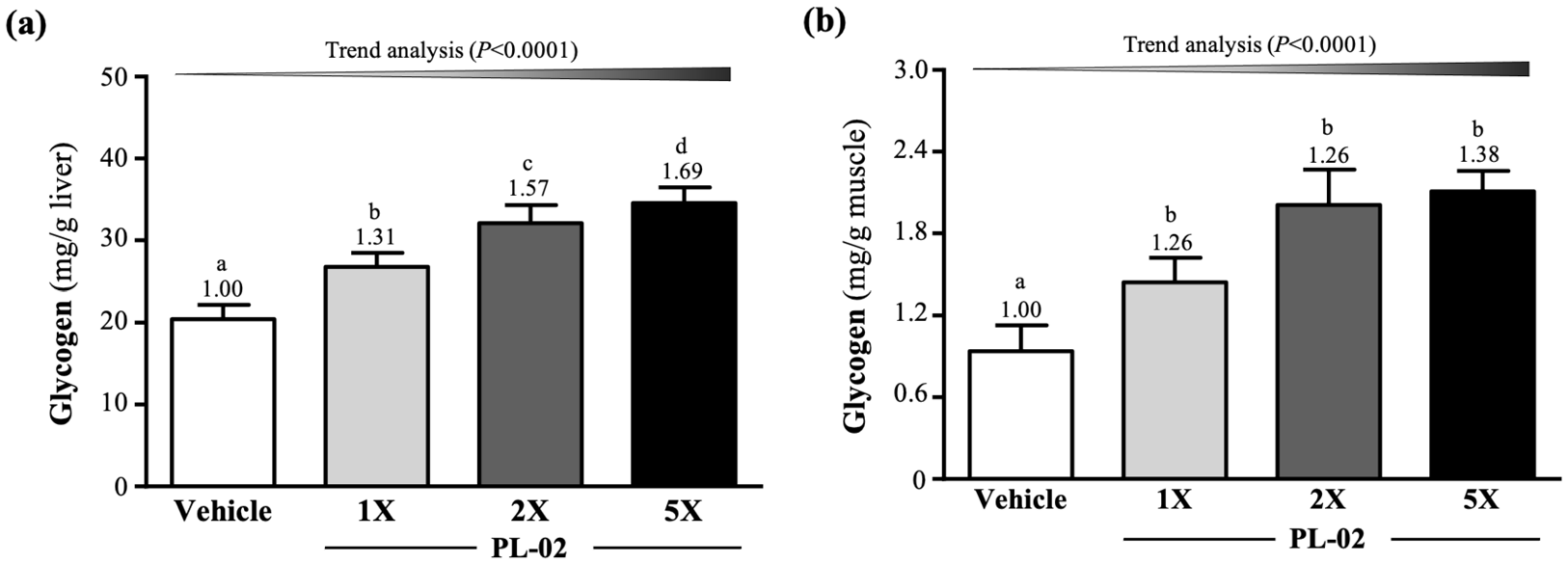
Effect of 4 weeks PL-02 supplementation on (**a**) liver glycogen and (**b**) muscle glycogen. Data are expressed as mean ± SD for n = 10 mice per group. Different superscript letters (a, b, c, d) indicate significant difference at *p* < 0.05.

The liver glycogen levels of the mice in the vehicle, PL-02-1X, PL-02-2X, and PL-02-5X groups were 20.44 ± 1.75, 26.75 ± 1.73, 32.09 ± 2.24 m and 34.58 ± 1.88 mg/g liver, respectively (Fig. 3a). Compared with the vehicle group, the PL-02-1X, PL-02-2X, and PL-02-5X groups were significantly improved by 1.31 -fold (*p* < 0.0001), 1.57 -fold (*p* < 0.0001), and 1.69 -fold (*p* < 0.0001), respectively. Muscle glycogen levels in the vehicle, PL-02-1X, PL-02-2X, and PL-02-5X groups were 0.94 ± 0.19, 1.44 ± 0.18, 2.01 ± 0.26 m and 2.11 ± 0.15 mg/g muscle, respectively (Fig. 3b). The PL-02-1X, PL-02-2X, and PL-02-5X groups were significantly increased by 1.53-fold (*p* < 0.0001), 2.14-fold (*p* < 0.0001), and 2.24-fold (*p* < 0.0001), respectively, as compared to the vehicle group. The effect of PL-02 supplementation on liver and muscle glycogen content was also dose dependent (*p* < 0.0001).

### Effect of PL-02 supplementation on biochemical profiles at the end of the study

We assessed whether supplementation of PL-02 for four weeks had an impact on the health and safety and conducted biochemical parameter tests (Table 3). As a result, all biochemical parameters were within the normal range. In addition, there were no significant differences between the groups. Therefore, we believe that supplementation with the doses with PL-02 will not cause any damage.

**Table 3:** Effects of PL-02 on biochemical parameters.

Parameter	Vehicle	PL-02-1X	PL-02-2X	PL-02-5X	Trend analysis
AST (U/L)	82 ± 7	81 ± 8	82 ± 7	82 ± 6	0.7425
ALT (U/L)	57 ± 18	55 ± 8	55 ± 8	56 ± 8	0.0367
CK (U/L)	563 ± 74	568 ± 73	564 ± 38	571 ± 61	0.7962
GLU (mg/dL)	228 ± 16	228 ± 38	226 ± 15	227 ± 51	0.7189
CREA (mg/dL)	0.39 ± 0.01	0.38 ± 0.01	0.38 ± 0.01	0.38 ± 0.03	0.0474*
BUN (mg/dL)	22.4 ± 2.5	22.8 ± 2.5	22.3 ± 2.8	22.8 ± 1.9	0.6891
UA (mg/dL)	1.3 ± 0.3	1.3 ± 0.2	1.3 ± 0.3	1.3 ± 0.2	0.5371
TC (mg/dL)	152 ± 8	152 ± 13	152 ± 20	152 ± 12	0.9640
TG (mg/dL)	157 ± 21	163 ± 39	156 ± 12	156 ± 30	0.9822
ALB (g/dL)	3.3 ± 0.1	3.3 ± 0.1	3.3 ± 0.1	3.3 ± 0.2	0.6818
TP (g/dL)	5.7 ± 0.3	5.7 ± 0.2	5.7 ± 0.2	5.7 ± 0.3	0.9014

Data are expressed as mean ± SD (n = 10 mice per group).

AST, aspartate aminotransferase; ALT, alanine transaminase; CK, creatine kinase; GLU, glucose; CREA, creatinine; BUN, blood urea nitrogen; UA, uric acid; TC, total cholesterol; TG, triacylglycerol; ALB, albumin; TP, total protein.

### Effect of PL-02 supplementation on tissue histology

**Figure 4:**
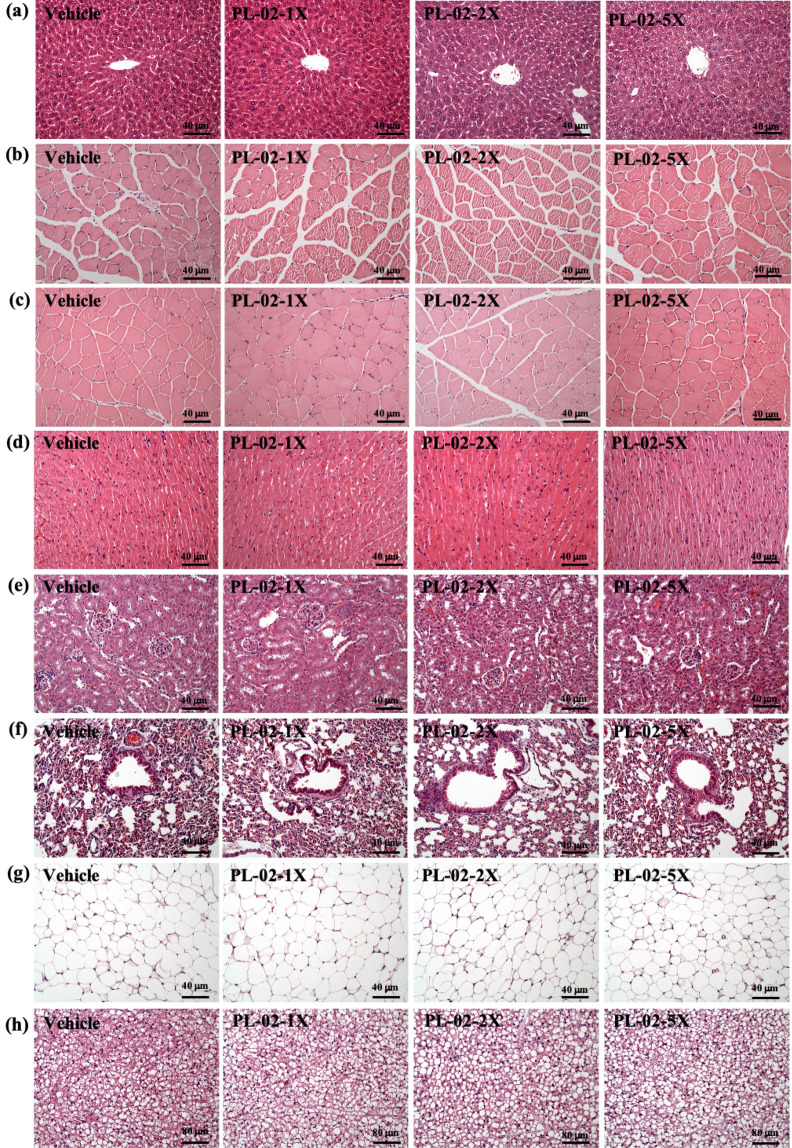
Effect of PL-02 supplementation on (**a**) liver, (**b**) muscle, (**c**) quadricep muscles, (**d**) heart, (**e**) kidney, (**f**) lung, (**g**) adipocyte tissue, and (**h**) BAT tissue in mice. (H&E stain, magnification: × 200; bar, 40 μm; BAT magnification: 100 × ; bar, 80 μm).

At the end of the study, we performed a histological evaluation of the liver, muscle, heart, kidney, lung, EFP, and BAT of the mice. As shown in Fig. 4, no abnormalities were observed in all groups. These results indicate that at the doses tested in this study, PL-02 has no adverse effects on organs and tissues.

### Effect of PL-02 Supplementation on gut microbiota

**Figure 5:**
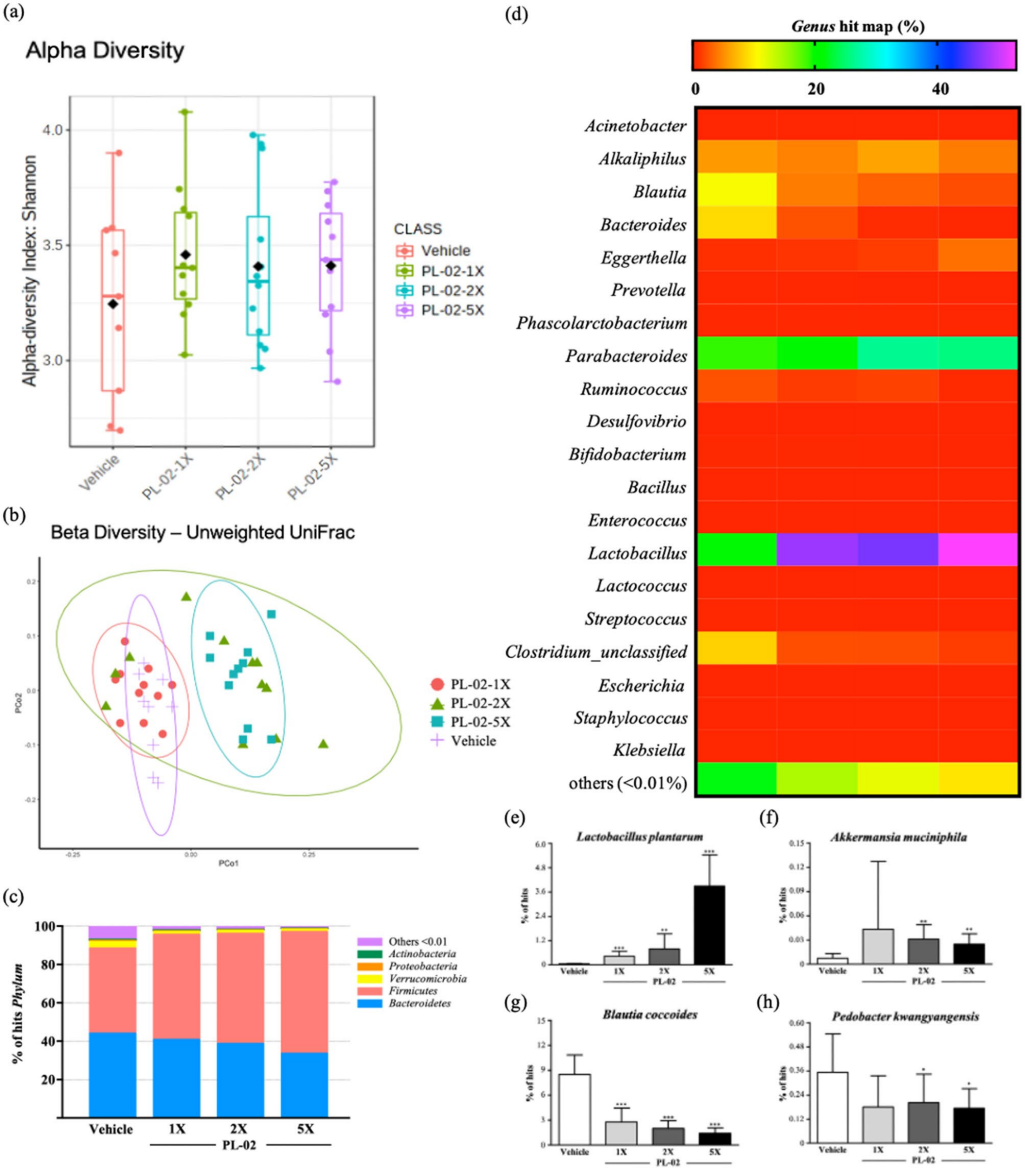
Effect of PL-02 supplementation on (**a**) alpha diversity, (**b**) beta diversity, (**c**) *phylum*, (**d**) *genus*, (**e**) *Lactobacillus plantarum* % hits, (**f**) *Akkermansia muciniphila. %* hits, (**g**) *Blautia coccoides % hits*, and (**h**) *Pedobacter kwangyangensis % hits*. Data are expressed as mean ± SD for n = 8 mice per group. Values with different superscript letters are significantly different at *p < 0.05; **p < 0.01; ***p < 0.0001.

At the end of the experiment, we analyzed the composition of the gut microbiota of mice treated with vehicle or PL-02 through 16S rRNA and observed the great changes in microbial ecology after PL-02 treatment. PL-02 did not significantly change alpha diversity (Shannon Diversity) (Fig. 5a), but it could change beta diversity by using Unweighted UniFrac model, which produced significantly different clusters between vehicle control and others (1X, 2X, and 5X, *p* = 0.013, *p* = 0.009 and *p* < 0.001, respectively, PERMANOVA by Adonis). (Fig. 5b). As shown on Fig. 5c, with the increased of the dose of PL-02, the percentage of *Firmicutes* in the gut microbiota also increased significantly. Among them, we could clearly see from the heat map that compared with the vehicle group (Fig. 5d), supplementation with PL-02 could effectively increase the proportion of *Lactobacillus* in the gut microbiota. With the increase in the dose of PL-02 supplementation, the proportion of *L. plantarum* (Fig. 5e) in the gut microbiota increased significantly (*p* < 0.0001), and the abundance of the specific gravity of *Akkermansia muciniphila* (Fig. 5f) also increased significantly (*p* < 0.01). In particular, the human harmful gut microbiota, *Blautia coccoides* (Fig. 5g) and *Pedobacter kwangyangensis* (Fig. 5h), were significantly lower in the PL-02-treated group than in the vehicle group, and the percentage of hits were inversely proportional to dose increase (*p* < 0.05).

## Discussion

In recent years, probiotics have been commonly used to promote health and improve body function, as well as for different benefits depending on the characteristics of the strain. In the current study, we screened the human-origin probiotic *L. plantarum* (PL-02) and designed different doses of probiotics to explore the benefits of improved exercise performance and anti-fatigue. We found that supplementing PL-02 for four weeks can significantly increase the proportion of health-associated bacteria in the intestine, muscle mass, muscle strength, endurance performance, and glycogen storage, and significantly reduce fatigue biochemical parameters after exercise, such as lactate, CK, and blood ammonia. In addition, it would not cause damage to healthy biochemical parameters and or cause tissue pathologies.

Supplementation with probiotics can effectively increase the α diversity in the intestinal and can also increase the production of SCFA in the intestine, thereby helping to maintain or restore the homeostasis of the intestine, promote health, and prevent many diseases^31^. Many studies have explored the crosstalk path between the gut and muscles and have found that the composition and interaction environment of the flora may affect the quality, function, and energy metabolism of the muscles by changing the flora, which is the so-called gut–muscle axis^32,33^. Among them, the metabolites of the gut microbiota may be involved in stimulating muscle energy metabolism and enhancing endurance performance, such as acetic acid^34^. Acetic acid enhances glucose uptake and fatty acid metabolism by activating the activated protein kinase (AMPK) and increasing the expression of glucose transporter type 4 (GLUT4) and myoglobin. The myocyte enhancer factor 2A (MEF2A), is a well-known transcription factor involved in the expression of myoglobin as well as GLUT4 genes and found in the treat cell. Therefore, it is considered an important metabolic pathway for increasing muscle mass^35^. In previous studies, Supplementing heat-inactivated Bifidobacterium breve B-3 for four consecutive weeks in rats found significant increases in muscle strength and mass, cytochrome C oxidase (CCO) gene expression, peroxisome proliferator-activated receptor γ coactivator-1α(PGC-1α) and phosphorylated AMPK in muscle^34^. In addition, previous study had shown that, 6-week supplementation of *L. plantarum* from kimchi has been found to significantly increase muscle mass in mice and humans^22,23^. These results seem to confirm that in this study, supplementation with human-origin *L. plantarum* also has significant benefits on improved muscle mass (Table 1) and strength (Fig. 1a,b).

On the other hand, propionic acid in SCFA can promote gluconeogenesis in liver cells^36^, while butyrate can maintain blood glucose homeostasis and promote glycogen metabolism through the GPR43-AKT-GSK3 signaling pathway^37^. In a previous study, it was shown that probiotics (*Lactobacillus acidophilus*) regulate glycogen synthesis-related genes (GSK-3β and Akt) and glycogen content in tissues^38^. In a previous study, researchers explored the correlation between the composition of the gut microbiota and the content of muscle glycogen. The results showed that germ-free mice had lower muscle glycogen levels compared to individuals with a normal microbiome composition^39^. The glycogen content in the liver and muscles is a key factor in determining the aerobic energy metabolism of athletes. Disturbances in glycogen levels may lead to insufficient energy supply and decreased muscle strength and function, leading to impaired bioenergy metabolism^40^. Optimizing and increasing glycogen storage can effectively improve exercise endurance performance, thereby delaying fatigue after exercise and accelerating recovery^41^. In addition, previous studies have confirmed that supplementation with probiotics can significantly increase SCFA and fatty acid oxidation and activate peroxisome proliferator-activated receptor γ coactivator 1α (PGC-1α), increasing ATP production, providing the energy needed for exercise and improving endurance exercise performance^42^. In our study, 4-week supplementation with *L. plantarum* PL-02 not only increased glycogen storage (Fig. 3), but also improved endurance exercise performance (Fig. 1c).

Similar to lactate, blood ammonia, BUN concentration, and CK activity would increase with the increasing time and intensity of exercise and would gradually recover with rest. Therefore, in many studies related to exercise and fatigue, they were often used as an important basis for judging peripheral fatigue caused by exercise^27^. Lactate was the product of glycolysis in the anaerobic energy system and was accompanied by the production of hydrogen ions. With the increase in hydrogen ion concentration, the decrease in pH in blood and muscle tissue would cause muscle contraction and glycolysis to be inhibited, which could easily lead to muscle damage and decreased exercise capacity. In addition, in the process of ATP resynthesis, when AMP deaminase (AMPD) deaminates adenosine monophosphate (AMP) to inosine monophosphate (IMP), ammonia is produced and accumulated in skeletal muscle. Ammonia could be metabolized into BUN through the urea cycle^30^. However, probiotics could improve the production of blood lactate during exercise and be converted into SCFA, especially propionic acid and butyric acid, as well as speeding up the conversion of butyric acid into acetyl-CoA, which is used to generate ATP in the Krebs cycle and then provide the energy needed during exercise^43^. On the other hand, probiotics could also reduce intestinal permeability and inhibit bacterial urease activity to reduce ammonia in the blood^44^. Therefore, these results seemed to be validated in our research. We found that supplementation with PL-02 for four consecutive weeks could not only reduce the blood lactate concentration and increase the rate of recovered after exercise, but also accelerate the elimination and recovery of blood lactic acid (Table 2) and reduced blood ammonia, BUN, and CK after exercise (Fig. 2a,c,d).

Through the 16S RNA analysis, we explored the distribution of gut microbiota by supplementation with *L. plantarum* (PL-02). The result showed that PL-02 supplementation could change the distribution ratio of the gut microbiota, which also seemed to play a role in energy absorption and regulation. A previous study showed that the core function of *L. plantarum* concerns carbohydrate metabolism and amino acid metabolism and provides molecular support for the strain to metabolize various sugars. Genes related to carbohydrate absorption and metabolism were closely related to genes encoding glycosidase and transporters^45^. Therefore, in our results it could be seen that as the feeding dose increases, the proportion of *L. plantarum* detected in the intestinal tract also increases significantly (Fig. 5e). In addition, we also found that compared with the placebo group, supplementation with PL-02 can significantly increase the proportion of *A. muciniphila* in the intestine (Fig. 5f). Previous studies have pointed out the higher proportion of the *A. muciniphila* genus in athletes’ intestines, compared with sedentary people. They could maintain the intestinal barrier function and glucose homeostasis and played a role when endurance exercise was impaired. Therefore, *A. muciniphila* is positively correlated with exercise performance^25^. In addition, the supplementation of PL-02 increases the proportion of *L. plantarum* in the intestine and also effectively reduces the proportion of *Bhatia coccids* and *Pedobacter kwangyangensis*. According to a previous study, *Blautia*, especially *B. coccoides*, may activate the secretion of tumor necrosis factor-α (TNF-α) and cytokines to a greater extent. *Blautia* is the most representative genus in type 2 diabetes patients and in prediabetes patients and is significantly higher than in patients with normal glucose tolerance^46^. According to another animal experiment, the abundance of *Pedobacter kwangyangensis* in the gut of mice on a high-calorie diet was greatly increased. Perhaps the abundance of this strain is related to obesity^47^. In the current study, we believe that supplementation with *L. plantarum* PL-02 for four consecutive weeks would not cause any physiological, biochemical, or histopathological hazards.

In the current study, in addition to functional performance and fatigue-associated biomarkers, we also needed to observe the changes in the gut microbiota and histopathological of the supplement with *L. plantarum*, which must be carried out through animal experiments. However, it is more difficult to use animal experiments to detect explosive power and other physical fitness tests related to short-term output. In addition, the understanding of the detailed mechanism warrants further study. In our previously report, whether the *L. plantarum* TWK10 selected from kimchi or the *Bifidobacterium longum* OLP-01 selected from weightlifters, they had been proven in animal and human experiments that they could effectively increase the proportion of strains in the gut microbiota, and also improved exercise performance and reducing exercise fatigue^22,23,30,48^. We estimate that PL-02 will have similar effects in future human trials. Moreover, the psychological and physical demands during strenuous exercise can trigger a stress response, activate the sympathetic nerve-adrenal medulla and hypothalamic–pituitary–adrenal (HPA) axis, leading to the release of stress and catabolic hormones, inflammatory cytokines and microbial molecules^49^. Therefore, this basic research also laid the foundation for future study, such as the combination with sports training, and as well as human trials.

In conclusion, we found that a 4-week supplementation with human-origin strain *L. plantarum* PL-02 isolated from weightlifting athletes could significantly improve grip and endurance exercise performance and increase muscle mass. Moreover, PL-02 could significantly reduce the level of fatigue indicators, such as lactate, BUN, ammonia, and CK. In addition, PL-02 would not cause harm on physiological performance or in tissue histopathology. Therefore, we believe that PL-02 could be used as a supplement to improve exercise performance and for its anti-fatigue bioactivity.
